# Aging Alters Olfactory Bulb Network Oscillations and Connectivity: Relevance for Aging-Related Neurodegeneration Studies

**DOI:** 10.1155/2020/1703969

**Published:** 2020-05-02

**Authors:** A. Ahnaou, D. Rodriguez-Manrique, S. Embrechts, R. Biermans, N. V. Manyakov, S. A. Youssef, W. H. I. M. Drinkenburg

**Affiliations:** ^1^Department of Neuroscience, Janssen Research & Development, a Division of Janssen Pharmaceutica NV, Turnhoutseweg 30, B-2340 Beerse, Belgium; ^2^Department of Non-Clinical Safety, Janssen Research & Development, a Division of Janssen Pharmaceutica NV, Turnhoutseweg 30, B-2340 Beerse, Belgium

## Abstract

The aging process eventually cause a breakdown in critical synaptic plasticity and connectivity leading to deficits in memory function. The olfactory bulb (OB) and the hippocampus, both regions of the brain considered critical for the processing of odors and spatial memory, are commonly affected by aging. Using an aged wild-type C57B/6 mouse model, we sought to define the effects of aging on hippocampal plasticity and the integrity of cortical circuits. Specifically, we measured the long-term potentiation of high-frequency stimulation (HFS-LTP) at the Shaffer-Collateral CA1 pyramidal synapses. Next, local field potential (LFP) spectra, phase-amplitude theta-gamma coupling (PAC), and connectivity through coherence were assessed in the olfactory bulb, frontal and entorhinal cortices, CA1, and amygdala circuits. The OB of aged mice showed a significant increase in the number of histone H2AX-positive neurons, a marker of DNA damage. While the input-output relationship measure of basal synaptic activity was found not to differ between young and aged mice, a pronounced decline in the slope of field excitatory postsynaptic potential (fEPSP) and the population spike amplitude (PSA) were found in aged mice. Furthermore, aging was accompanied by deficits in gamma network oscillations, a shift to slow oscillations, reduced coherence and theta-gamma PAC in the OB circuit. Thus, while the basal synaptic activity was unaltered in older mice, impairment in hippocampal synaptic transmission was observed only in response to HFS. However, age-dependent alterations in neural network appeared spontaneously in the OB circuit, suggesting the neurophysiological basis of synaptic deficits underlying olfactory processing. Taken together, the results highlight the sensitivity and therefore potential use of LFP quantitative network oscillations and connectivity at the OB level as objective electrophysiological markers that will help reveal specific dysfunctional circuits in aging-related neurodegeneration studies.

## 1. Introduction

Aging is the leading risk factor, which promotes a large class of neurodegenerative brain diseases that are particularly feared such as Alzheimer's disease (AD) and Parkinson's disease (PD) [1–3]. As the brain ages, both functional and structural changes result in a natural slow decline of cognitive processes. The aging impacts functional aspects of all the organs and biological pathways resulting in protein aggregations, early synapse damage, and selective neural network dysfunction. These changes make the brain more susceptible to augmenting amyloidogenic metabolism of APP, promoting the toxicity of A*β* oligomers, enhancing the hyperphosphorylation of tau, and accelerating the formation of neurofibril tangles or synucleopathy [4–6]. Other changes associated with loss of brain volume include atrophy of white matter, accumulation of damage in nuclear and mitochondrial DNA, epigenetic alterations, mitochondrial dysfunction, loss of proteostasis, stem cell exhaustion, and altered intracellular communication and neuroinflammation [5].

The process of aging may have detrimental effects on neural activities, leading to a breakdown in critical synaptic plasticity and connectivity. Synaptic plasticity, which is the ability of synapses to strengthen (long-term potentiation LTP) and weaken (long-term depression LTD) over time in response to increases and decreases in their activity, plays a major role in the persistent long-term changes associated with learning, memory, and cognitive functions. The intricate balance between LTP and LTD processes plays a major role in the encoding and storage of new, incoming information [7]. The natural processes of aging damage and destroy synaptic connectivity, leading to a decline in normal synaptic plasticity mechanisms and providing a likely neural basis for the decline in memory and cognition associated with age [8].

The hippocampus, the epicenter of memory function and a highly plastic structure, is the first to degenerate in both normal aging and AD/PD brains, leading to a breakdown in the trisynaptic circuit, in which the CA1 region is a key part. Atrophy of these areas will lead to a significant decline in synaptic transmission and plasticity within the trisynaptic circuit and detrimental to declarative and spatial memory functions which rely on synaptic plasticity to consolidate and encode new incoming information [9]. This process is heightened in AD/PD patients, accounting for the symptomatic loss in memory. The age-related impairments in memory processes have been linked to modulation of activity-dependent forms of synaptic plasticity (Schaffer collateral/commissural fiber synapses) in the CA1 region of the hippocampus. This LTP is typically induced by brief trains of high-frequency synaptic stimulation (HFS), which is dependent on NMDA receptor activation and synapse specificity. Several studies have described deficits in activity-dependent forms of synaptic plasticity in the hippocampal CA1 region of aged animals, which is believed to be underlying physiological mechanisms that might represent a physiological basis for age-dependent deficits in memory. Most of those studies have used induction protocols to examine whether LTP is altered in hippocampal slices from aged animals [10–13].

However, a growing view argues against ubiquitous neuronal loss and brain atrophy during aging [14, 15]. Accordingly, subtle regional abnormalities of neural structure and synaptic connectivity were observed in the sensory cortex and hippocampus, which may be underlying cognitive decline associated with aging. Sensory olfaction processing such as odor identification, odor memory formation, and discrimination is sensitive to aging [16–19]. Ultrastructural observations showed that the cellular organization of the olfactory bulb remained stable during aging; however, discrete region and layer-specific neuronal loss were revealed in the glomerular layer resulting in a disorganized olfaction function [20].

The waking electrooculogram (EEG) in aged mice is characterized by slowing of the theta frequency rhythm, which is a prevalent activity during exploratory motor activity events [21]. The leftward shift of high frequency to slow EEG oscillations has been confirmed in epidural cortical recordings of passive waking in aged mice [22]. Higher theta and gamma frequency activities have been related to higher cognitive functioning, learning, and memory processing [23–25], whereas lower theta and gamma network oscillations are two hallmarks associated with aging-related cognitive deficits [26–28].

The present study used young adult and aged wild-type C57Bl/6 mice to describe the circuit-level mechanisms of age-related deficit in higher cognitive functioning. The olfactory bulb and hippocampus were chosen because of their wide connectivity and role in spatial learning and memory and odor sensory processing, which are compromised by aging.

## 2. Materials and Methods

### 2.1. Animals

All experiments were performed under strict adherence with the guidelines of the Association for Assessment and Accreditation of Laboratory Animal Care International (AALAC) and with the European Communities Council Directive of 22^nd^ September 2010 (2010/63/UE) and were approved by the local ethical committee. Male C57BL/6 mice (Bl6) of 3 and 22-23 months (*n* = 16 in total: *n* = 8 young and *n* = 8 aged animals) obtained from Janvier were group housed with their litter mates in individually ventilated cages at a relative humidity of 55% ± 10 and 22°C ± 2 temperature. They were kept at a 12 h/12 h light/dark cycle and had standard food and water available ad libitum.

### 2.2. In Vivo Local Field Potential (LFP) Procedures

#### 2.2.1. Surgery

Surgery was carried out in animals weighing between 20 g and 28 g at the time of electrode implantation. Animals were anesthetized with isoflurane and injected with piritramide (0.25 mg/kg). They were mounted in a stereotaxic frame (David Kopf Instruments), with the incisor bar around 5 mm beneath the centre of the ear bars. This was adjusted to ensure that the height of the skull surface was equal to bregma and lambda, according to the stereotaxic mouse brain atlas of Paxinos and Franklin [29]. A heating pad was placed under the animals to maintain their core body temperature at 38°C. Animals were then stereotaxically equipped with 8 stainless steel recording electrodes in the olfactory bulbs (OB) (AP: +4.3 mm from Bregma, ML: ±1.2 mm, DV: -2 mm), frontal cortex (AP: +2 mm from Bregma, ML: ±1.5 mm), the lateral entorhinal cortex (EC) (AP: -2.9 mm from Bregma, ML: -4 mm, DV: -4.5 mm), and the ventral hippocampus CA1 (AP: -1.7 mm from Bregma, ML: -1.5 mm, DV: -1.7 mm), the dorsal hippocampus CA1 (AP: -1.94 mm from Bregma, ML: +1 mm, DV: -1.25 mm), and the lateral amygdala (AP: -2.06 mm from Bregma, ML: +3.25 mm, DV: +3.2 mm). All electrodes were referenced to a ground screw electrode, placed above the midline of the cerebellum. Electrodes were connected to a pin with a small insert (Future Electronics: 0672-2-15-15-30-27-10-0) (Track pins; Dataflex: TRP-1558-0000) and were inserted into a 10-hole connector, which was carefully fixed to the skull with dental cement.

#### 2.2.2. Experimental Design, Recording, and Analysis

After one-week recovery period and adaptation to recording conditions, EEG were recorded for 20 hours during the dark phase of the circadian cycle, under vigilance-controlled wake, as described elsewhere [30, 31]. Recordings were taken in the animal's home cages placed in a sound-attenuated Faraday cages. Motor activity was measured by a pair of passive infrared (PIR) detectors located above every recording cage. Motion levels were analyzed from the envelope of activity from both PIR detectors. Continuous EEG recordings were acquired with Biosemi ActiveTwo system (Biosemi, Amsterdam, Netherlands). Analogous signal was band-pass filtered between 1 and 256 Hz at a sampling rate of 512 Hz with a range of ±500 mV.

#### 2.2.3. EEG Spectra

EEG recordings were derived from eight brain regions under vigilance waking condition during the dark circadian phase. Artifact-free waking epochs with high to moderate body activities were considered in the analysis. Epochs with high-voltage slow cortical waves in the absence of locomotor activities were discarded. Powerline inference was removed using a filter described in the supplementary materials in [32]. Analysis was performed using a custom-made scripts in MATLAB toolbox described earlier [30, 31]. Briefly, spectral power density was calculated in 2 second sliding windows using a Welch's method with Hanning window, and the power spectra was expressed as relative power for each frequency over 1-256 Hz. Average across recording time relative power in each frequency bin of each location was averaged across animals for young and aged mice separately to visualize the grand averaged relative spectra. One young mouse was discarded from the analysis due to artefacts in the EEG signals. For the sake of clarity in presenting spectral data, graphs only shown the frequency range between 1 and 7 Hz and from 30 to 80 Hz, and box plots only show results for delta (1-4 Hz) and gamma (30-80 Hz) bands.

#### 2.2.4. Coherence

Longitudinal effects of aging on the integrity of cortical neural pathways and the functional coupling between different cortical structures at various frequency oscillations were estimated according to the procedure described earlier [30, 31]. The coherency function, which gives information on the stability of the similarity at each frequency bin between the time series in different electrodes, was quantified at different time points by the normalization of the cross-spectrum by the square root of the product of the autospectra: Coherency(*f*) = S_AB_(*f*)/sqrt(S_AA_(*f*)S_BB_(*f*)), where S_AB_ is the cross-spectrum between the signals A and B, S_AA_ is the autospectrum of the signal A, and S_BB_ is the autospectrum of the signal B. To characterize the strength of the interaction between signals A and B, magnitude-squared coherence was used. It is quantified as Coh(*f*) = |Coherency(*f*)|^2^ and is reported as normalized values between 0 and 1: A low value indicates no similarity between the two signals, whereas values close to 1 indicate a high similarity between two time series signals up to near constant phase shift. It is known that coherence estimation is highly influenced by volume conductance effect [33]. To reduce volume conductance effect, an imaginary part of coherency was used [34].

#### 2.2.5. Phase-Amplitude Cross-Frequency Coupling

To estimate whether high frequency EEG amplitudes are modulated by low frequency phase variations for the same electrode site signals, phase-amplitude coupling (PAC) was calculated using the algorithm based on modulation index (MI) [35, 36]. MI is estimated as a mean (a long time *t*) absolute value of the signal *z*(*t*) = A_H_(*t*)∙exp(i∙*φ*_L_(*t*)), $i=\sqrt{-1},$ using instantaneous phase *φ*_L_(*t*) around low frequency *f*_L_, and instantaneous amplitude envelope A_H_(*t*) around high frequency *f*_H_. In order to extract time-varying frequency band-specific amplitude A_H_(*t*) and phase *φ*_L_(*t*), a raw nonfiltered EEG signal was convoluted with complex Morlet wavelets. For PAC estimation, *f*_L_ was varied in an interval of 2-12 Hz with a step of 2 Hz, and all *f*_H_ taken from interval 10-200 Hz with a step of 5 Hz were considered.

### 2.3. *In vivo* Long-Term Potentiation (LTP) Procedures

#### 2.3.1. Anesthesia

Mice were anesthetized using sodium pentobarbital [37–39]. The mice received a first bolus intraperitoneal administration of the anesthetic, at 60 mg/kg formulated in H_2_O and NaCl solvent and after 10 min a second bolus of 10 mg/kg. Throughout the experimental procedure, mice were placed on the heated pad, and the depth of anesthetic state was checked by pedal reflex and was maintained by hourly administration of the anesthetic (10 mg/kg, 0.1 ml for 10 g body weight).

#### 2.3.2. Surgery

Following observation of an adequate depth of anesthesia, the mice were placed in a stereotactic frame. Core body temperature was maintained using a thermostatically controlled heating pad. An incision was made along the midline of the head, and bregma was defined. Additionally, it was checked that the skull was not tilted at any axe. A bipolar stimulating electrode of tungsten wire with 0.5 M*Ω* impendence and 1-2 *μ*m tip diameter (World Precision Instruments) was inserted into the Schaffer collateral and a monopolar recording electrode of Teflon-coated tungsten wire with a 75 *μ*m outer diameter (World Precision Instruments) was inserted into the stratum pyramidal layer of the CA1. The coordinates for the Schaffer collateral are AP: -2,0; ML: -2,0; and DV: ~1.2 and for the stratum pyramidal layer are AP: -1,7; ML: -1,5; and DV: ~1.1 from dura. Two holes were drilled to insert reference and ground screws.

#### 2.3.3. Basal Synaptic Activity

Single square pulses (200 *μ*s, 3000 mV) were delivered, while descending the recording and stimulating electrodes (at 0.2 mm/min), to confirm their location in the brain. Labview homemade oscilloscope software was used to visualize the evoked field excitatory post synaptic potentials (fEPSPs). The electrode's dorsal-ventral position was measured from the dura before piercing it with a small needle.

Recordings of fEPSPs were made from the stratum pyramidale in the CA1 area of the right hippocampal hemisphere in response to stimulation of the ipsilateral Schaffer collateral-commissural pathway. Axon excitability was tested by generating an input/output curve that measured the amplitude of the curve compared to the slope of the fEPSP responses across a range of stimulation voltages.

### 2.4. Inclusion and Exclusion Criteria

fEPSPs recorded in the Labview oscilloscope software need to meet the inclusion and exclusion criteria prior to engaging in an input/output (I/O) curve: The latency to peak negative deflection of fEPSPs is within 6-10 ms, the maximum amplitude between 1500 *μ*V and 2500 *μ*V and its maximum slope must lie between 400 *μ*V/ms and 900 *μ*V/ms at 200 *μ*s stimulus duration. Once the response met the preset criteria, a functional input/output (I/O) curve is generated using neuroscience measurement custom-designed software. Stimulation at intensities ranging from 1000 to 10000 mV in steps of 1000 mV at 0.033 Hz frequency and 200 *μ*s duration was delivered, and 3 responses were recorded at each intensity. The curve was drawn using the mean of the 3 responses at each time point. The stimulus which evoked an fEPSP slope of 50% of the maximum response was selected as a test stimulus for the LTP induction procedure. All I/O curves followed a sigmoid curve distribution, and the calculated test stimulus fits between 3300 mV and 4700 mV for all experiments.

#### 2.4.1. LTP Induction

The procedure consists of calculating the change in magnitude of the evoked response at constant test stimuli before and after high frequency stimulation. Stimulation settings are put into neuroscience measurement custom-designed software and sent to the stimulation electrode via a data acquisition board linked to a constant current isolator unit (Multichannel System MC SRG4002). EEG and fEPSPs are recorded using a Biosemi Active Two amplifier (Differential amplifier, Netherlands) at a sample rate of 3 kHz. For each time point measured during the experiment, five records of evoked responses at the frequency 0.033 Hz and 200 *μ*s duration were averaged. The duration of the experiment is 30-60 min baseline followed by 90-120 min of posttetanisation. The last 30 min of the baseline recording (6 time points) was averaged and used as control for LTP induction. Custom-made analysis toll software normalizes data points and expresses them as a percentage of the last 30 min of baseline.

The high frequency stimulation (HFS) protocol that was used to induce LTP response, consisted of two trains of 50 pulses at 200 *μ*s pulse duration with an intertrain interval of 30 s and 100 Hz frequency [40–42].

### 2.5. Histological Marker of DNA Damage

After euthanasia, olfactory bulbs (OB) were collected from the brains of four young and four aged mice and fixed immediately in 10% neutral buffered formalin. Fixed tissues were processed with routine paraffin embedding technique and stained immunohistochemically with antibody (Anti-gamma H2A.X. Cat No. ab26350, Abcam, Cambridge, United Kingdom) against H2AX (histone 2 a family X), which is a standardized marker for DNA damage [43] and double stranded breaks at dilution of 1/500 (2 *μ*g/ml).

### 2.6. Data Analysis

Result for described EEG metrics and for groups of aged and young Bl6 mice are presented as mean values with 95% confidence intervals (CI). Between group difference in means was assessed using two-sample *t*-test, and in case of significance, it is indicated by asterisks on box plots (∗*p* value < 0.05, ∗∗*p* value < 0.01). In order to deal with multiple comparisons and account for strong relations of estimates at neighbouring frequencies, for spectra and coherence data, threshold-free cluster enhancement (TFCE, H = 2, E = 0.5, *α* = 0.05) was also used [44], which is based on t-statistics and provides threshold-independent advancement to cluster permutation test [45].

All LTP data were expressed as percentage of change from baseline and were presented as means ± SEM%. The slope of the fEPSP was calculated from least square linear fit performed on the 80% interval between the artefact end and the negative peak. fEPSP slopes were obtained every 2.5 min as an average of 5 responses at 0.033 Hz and were then expressed as mean percentage change from baseline (defined as the last 30 min prior tetanisation) ± SEM. Analysis of variance (ANOVA) followed by a post hoc test (Dunnett's test) were used to correct for multiple comparisons. Difference in means between groups was considered significant, if *p* value is below 0.05.

## 3. Results

### 3.1. Histology

**Figure 1:**
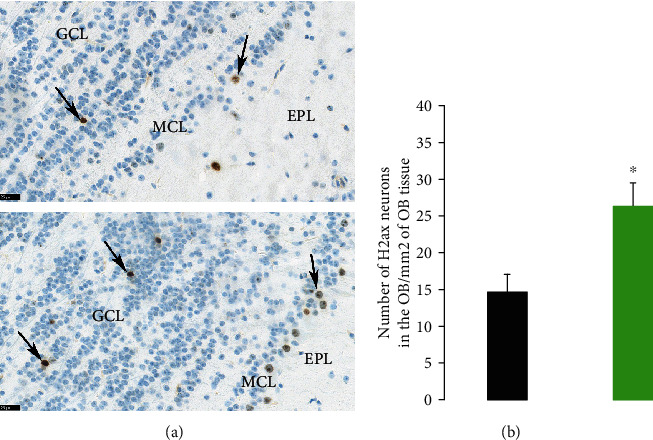
(a) Histological overview of the olfactory bulbs (OB) from 1 young mouse (top panel) and old mouse (bottom panel) stained for H2AX and taken at same magnification (scale bar = 25 microns). The OB of the old mouse exhibited significant increase in the numbers of H2AX-positive neurons particularly in the mitral cell layer (MCL) and the granular cell layer (GCL). H2AX-positive neurons are depicted by arrows. EPL: external plexiform layer. (b) Numbers of H2AX neurons in the olfactory bulbs of young and aged mice/mm^2^ of olfactory bulb tissue. Data are presented as mean values ± SEM for young (black, *n* = 4) and aged C57BL/6 mice (green, *n* = 4). Two-sample *t*-test.

The olfactory bulbs of older mice exhibited mild increase in the numbers of H2ax-positive neurons when compared to the OB of younger mice as shown Figures 1(a) and 1(b). The few positive H2AX-neourns in the OB of young mice were present mainly in the granular cells layer (Figure 1 top panel); however, in the OB of older mice, they were mainly present in the mitral cell layer and to lesser extent the granular cell layer (Figure 1 bottom panel).

#### 3.1.1. Attenuation of Wake LFP Gamma Oscillations in Aged Mice

**Figure 2:**
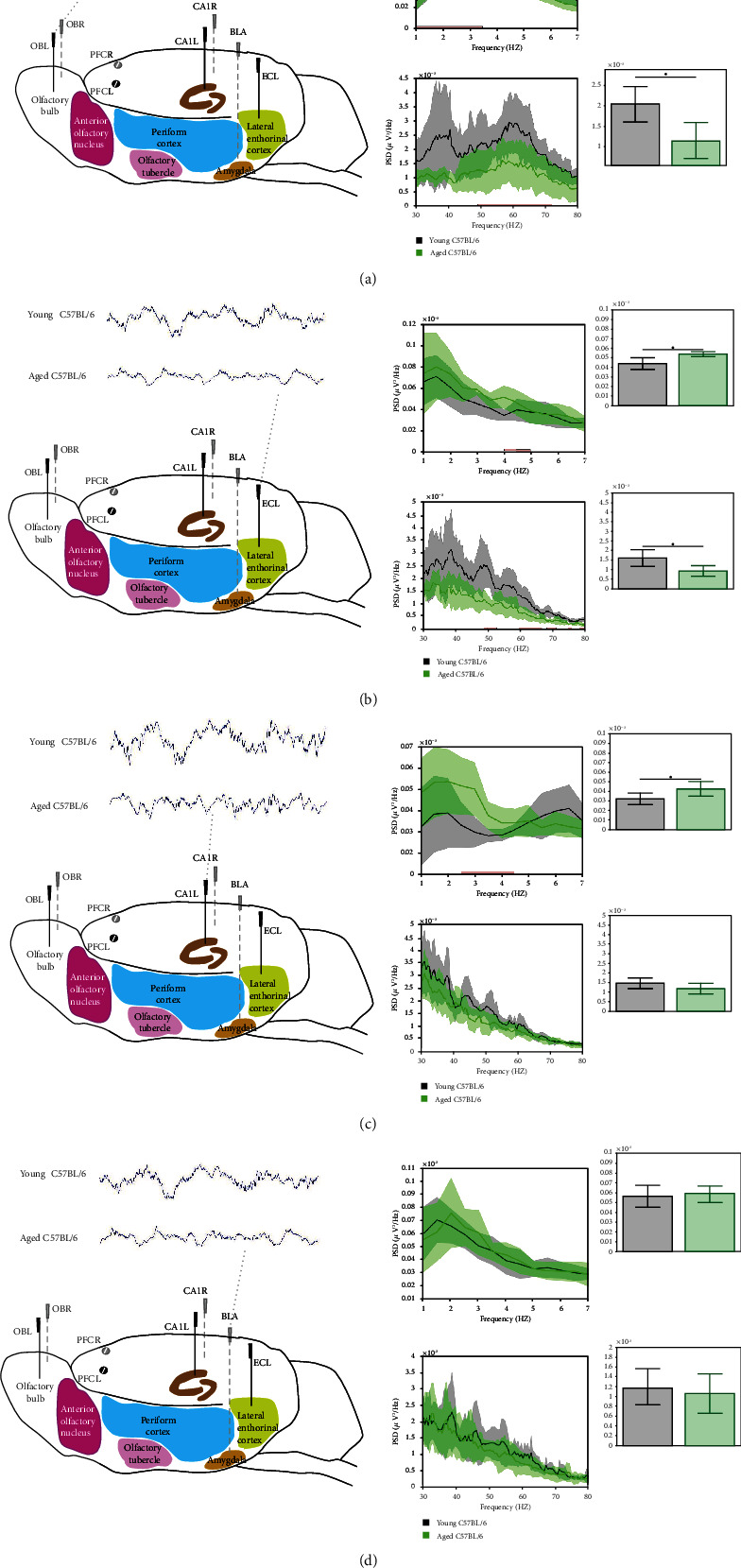
Relative power spectra data of wake LFP signals in low (1-7 Hz) and high (30-80 Hz left panels) frequencies for recordings performed in left hemisphere. (a) Olfactory bulb (OBL), (b) entorhinal cortex (ECL), (c) hippocampal CA1 (CA1L), and (d) basolateral amygdala (BLA) for young (black, *n* = 7) and aged (green, *n* = 8) C57BL/6 mice. Data are presented as mean values ± 95%confidence intervals for young (black, *n* = 7), and aged C57BL/6 mice (green, *n* = 8). Bars on the horizontal axis indicate clusters, which drive significant between group differences using threshold-free cluster enhancement (TFCE, *α* = 0.05). Right bar and whiskers panels show relative power in delta frequency 1-4 Hz (top) and gamma frequency 30-80 Hz (bottom). Lines above bar plots with asterisks indicate presence of significant between group difference, ∗*p* value < 0.05 (two-sample *t*-test), ∗*p* value < 0.05.

Wake LFP recoded from the OB of both young and aged animals showed a peak in the gamma frequency oscillations; however, aged mice showed significant reduction in relative power at cluster correspondent to this peak, driven by frequencies between 48 and 73 Hz (threshold-free cluster enhancement [TFCE] analysis) (Figure 2(a), bottom middle curve plot), which also could be seen using more traditional two-sample *t*-test for a total relative gamma power in the interval of 30-80 Hz (*p* = 0.01, Figure 2(a), bottom right bar plot). Using TFCE analysis at the EC network, a decrease with age in the relative power was found at clusters, driven by frequencies between 49-54 Hz, 60-67 Hz, 68-72 Hz, 75-76 Hz, and 78-80 Hz (Figure 2(b), bottom middle curve plot), which could also be seen in a reduction in total relative gamma power in the interval of 30-80 Hz with two-sample *t*-test (*p* = 0.02, Figure 2(b), bottom right bar plot).

No significant alteration was found in the gamma oscillatory pattern in the CA1 and BLA areas (Figures 2(c) and 2(d), bottom middle curve and right bar plots, respectively).

#### 3.1.2. Attenuation of Theta Oscillations and Shift towards Slow Rhythm Oscillations in Aged Mice

The age-dependent alterations in network oscillations appeared in the shift of the relative power into slow oscillatory rhythm (TFCE analysis with difference driven by oscillations below 3.5 Hz) at the OB network (Figure 2(a), top middle curve plot), which could also be identified by two-sample *t*-test in delta band (*p* = 0.03, Figure 2(a), top right bar plot). Increases in total relative power of the delta band were also observed in the EC area (*p* = 0.04, two-sample *t*-test; and correspondent TCFE analysis) (Figure 2(b), right top bar plot). Spectral analysis of LFP signals revealed that the activity in the theta frequency range, a correlate of arousal, was different between groups in the CA1 area (Figure 2(c), top middle curve plot), suggesting that aging also reduces vigilance during the active period. No alteration was observed in the slow frequency oscillatory pattern at BLA network (Figure 2(d), top middle curve and right bar plots).

#### 3.1.3. Attenuation of Theta-Gamma Phase-Amplitude Coupling in Aged Mice

The functional relevance of temporal interaction between superimposed network oscillations, estimated by the strength of cross-frequency coupling between the phase of slow and the amplitude of fast oscillations (PAC), has been associated with information processing in the brain. We examined whether aging altered the relationship between the gamma amplitude and the theta phase. The estimated mean PAC values in different recording sites at the OB and CA1 regions are qualitatively shown in the form of comodulation heat maps for young and aged mice.

**Figure 3:**
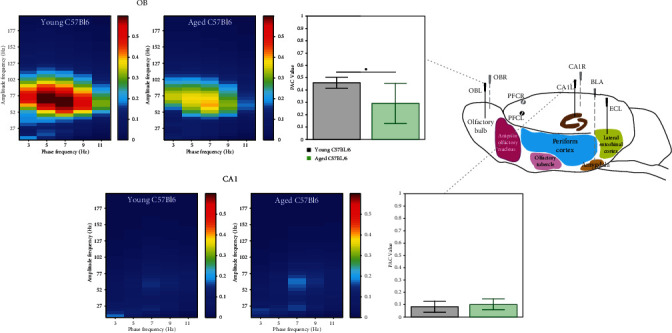
Heat maps showing the mean phase amplitude coupling (PAC) modulation index at the OB and CA1 recording electrodes for young (left panels in each frame) and aged (right panels in each frame) C57BL/6 mice. As shown by the color scale, “hotter” colors indicate high coupling values while “colder” colors indicate low or no coupling. Bar graphs showing the mean (across animals) theta-gamma PAC (with 95% CI) at the OB, EC, and CA1 electrodes for young (black, *n* = 7) and aged (green, *n* = 8) Bl6 mice. These means along animals' PAC values were computed as the average PAC for the large window of phase frequency: 2–12 Hz, and amplitude frequency: 10–200 Hz. Right bar charts show estimated mean PAC index phase 4-8 Hz and amplitude 40-100 Hz, and horizontal lines above bar plots with asterisks indicate presence of significant difference between genotypes (∗*p* value < 0.05). Data are presented as mean values (with 95% CI).

As depicted in Figure 3, top left panel, young mice animals exhibit high phase-amplitude coupling in the OB region. This high coupling peaks around a phase frequency of 7 Hz and amplitude frequency of around 57.5 Hz, in the theta-gamma range. Aged mice demonstrate reduced PAC in the OB area, whereas PAC comodulation heat maps in the CA1 areas of aged mice appear to be not affected (Figure 3, bottom panels). Quantification in the form of bar charts showed a significantly (*p* < 0.05, two-sample *t*-test) reduced mean theta-gamma PAC in the OB region in the aged group as compared to young mice (Figure 3, top right panels), respectively. The strength of theta-gamma coupling was weak in the CA1 area and did not differ between aged and young mice (Figure 3, bottom panels).

#### 3.1.4. Attenuation of Coherent Activity in the OB Area in Aged Mice

**Figure 4:**
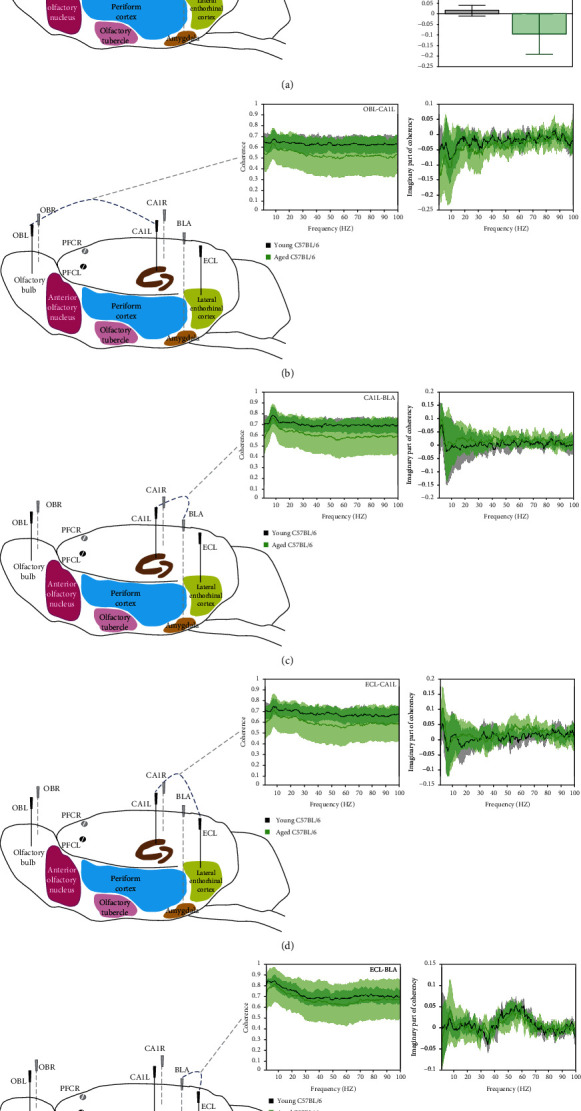
Coherence and imaginary part of coherency patterns between recording pairs (a) olfactory bulb left and right (OBL-OBR), (b) olfactory bulb left and CA1 left (OBL-CA1L), (c) CA1 left and basolateral amygdala (CA1L-BLA), (d) enthorinal cortex left and CA1 left (ECL-CA1L), (e) enthorinal cortex left and basolateral amygdala (ECL-BLA), in young (black, *n* = 7) and aged (green, *n* = 8) C57BL/6 mice. Graphs show mean values (±95% CI) as a function of frequency in 1-100 Hz range. Bars on the horizontal axis indicate clusters, which drive significant between group differences using threshold-free cluster enhancement (TFCE, *α* = 0.05). In panel (a), bar and whiskers panels show imaginary part of coherency for interval 25-35 Hz. Lines above bar plot with asterisks indicate presence of significant difference between groups, ∗*p* value < 0.05 (two-sample *t*-test).

We addressed whether aging affects network connectivity by measuring pairwise coherence and imaginary part of coherency in different networks during waking state (Figures 4(a) and 4(b)). Using threshold-free cluster enhancement (TFCE), we found significant between group difference in imaginary part of coherency in olfactory networks, driven by frequencies between 25 and 38 Hz (top right panel), which is also visualized in box plots for frequency interval 25-35 Hz (*p* = 0.04, two-sample *t*-test) (Figure 4(a)). No significant differences were found in OBL-CA1L (Figure 4(b)), CA1L-BLA (Figure 4(c)), ECL-CA1L (Figure 4(d)), and ECL-BLA (Figure 4(e)).

#### 3.1.5. Impairment of LTP Response to HFS in Aged Bl6 Mice

**Figure 5:**
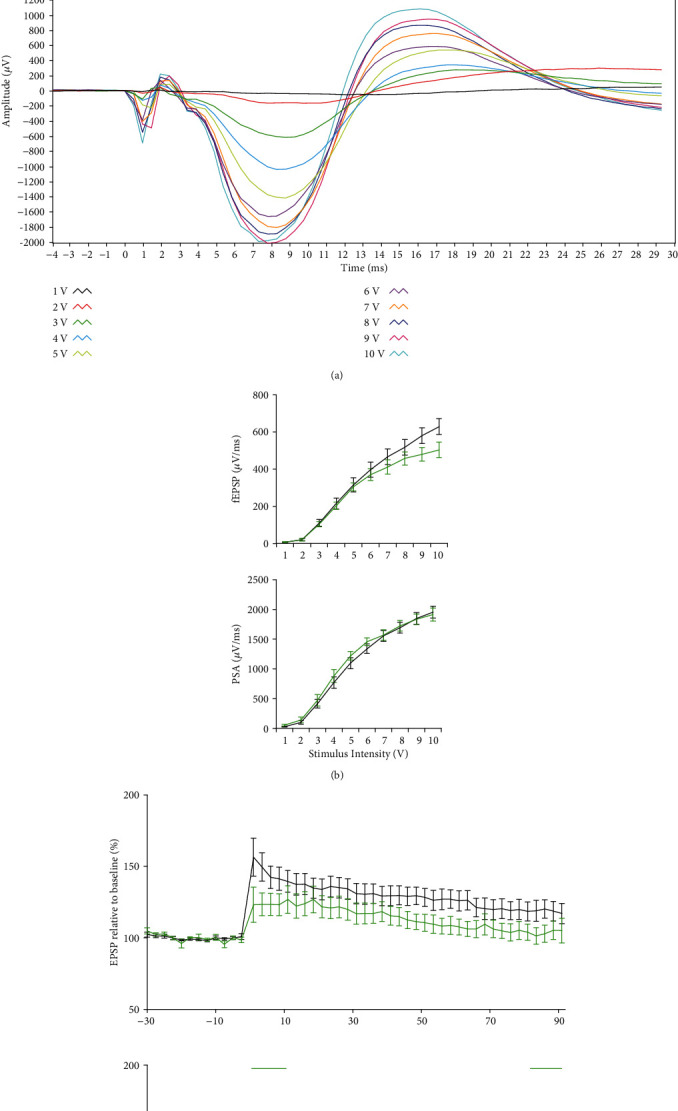
(a) Placement of the stimulation-recording electrodes at the Schaffer collateral-CA1 stratum pyramidal synapses in anesthetized mice (top panel), and a typical waveform spaghetti curves where the latency to peak negative deflection of fEPSPs is within 6-10 ms and the maximum amplitude between 1500 *μ*V and 2500 *μ*V. (b) Collective I/O curves of stimulation voltage and fEPSP slope (top panel) or PSA (bottom panel) values relative to baseline are plotted for young (black, *n* = 9) and aged (green *n* = 8) C57BL/6 mice. There was no significant alteration in baseline synaptic response, (c) A decline in synaptic response to HFS was observed in normalized EPSP and PSA values and was maintained throughout the recording session. No difference was observed in the average wave form during the 30 min baseline interval prior tetanisation, whereas smaller waveform was observed in aged mice during 0-30 min and 60-90 min posttetanisation. Data are presented as means ± SEM (%). Line above indicates statistical significance between groups, repeated ANOVA.

To examine whether aging affects plasticity mechanisms, we studied the plasticity response to HFS at the Shaffer collateral-CA1 stratum pyramidal synapse (Figure 5 top scheme), in aged and young C57BL/6 mice (Figure 5(a)). As revealed by I/O curves of fEPSP responses, a slight decrease was observed in basal synaptic activity of aged mice; however, there was no significant differences between the study groups (Figure 5(b) top panel). Similarly, no difference was observed in collective I/O curves of PSA, suggesting no overall difference in the basal synaptic activity between the study groups (Figure 5(b)bottom panel). However, aged mice exhibited declines in both fEPSPs and PSA values, suggesting impairments in the synaptic strength in response to HFS stimulation (Figure 5(c), top and bottom panels). A borderline significant difference was observed in fEPSPs of the early- and late-LTP phases (-33% and -18%, *p* = 0.05). PSA response values showed significant differences in early phase (-38%; *p* = 0.02) and late phase of LTP (-20.5%; *p* = 0.02) (Figure 5(c).

## 4. Discussion

The data of the present experiments confirm the age-associated deficits in plasticity mechanisms. We demonstrate that aged C57BL/6 mice exhibited significant increase in DNA damage as a marker of neurodegeneration, deficits in HFS-induced changes in synaptic strength as compared to young aged mice. The results further extend previous studies by showing age-related abnormalities in network oscillations and connectivity patterns that were particularly pronounced at OB circuit.

### 4.1. Aged Mice Exhibit Deficits in Hippocampal LTP Response to HFS, While no Alteration Was Observed in Basal Synaptic Activity

Aging plays a central role in age-related cognitive deficits [46], where synapses are particularly vulnerable sites of attack. Most mechanistic studies of memory storage have focused on the hippocampus, due to its critical role in learning and memory [47]. There is a high degree of overlap between the atrophy and degeneration of brain structures seen in normal aging and of that seen in AD brains [5]. The hippocampus is an early target for neurodegeneration and atrophy, which could be down to hippocampal hyperactivity and overactivation, with the CA1 subfield being one of the main targets for degeneration.

LTP has been used as an experimental model for studying mechanisms of memory and brain plasticity. It has been suggested that age-related impairments in learning and memory may be due to age-related deficits in LTP of glutamatergic synaptic transmission. Age-dependent effects on synaptic function are regionally heterogeneous [46]. In old rats, the synaptic density declines in the dentate gyrus, but not in the CA1, and electrophysiological studies using minimal stimulation suggest declines of the basal synaptic potency in CA1 but not in the dentate gyrus of aged animals [48]. In young animals, NMDA receptor-dependent LTP in the stratum radiatum of hippocampal area CA1 is thought to underlie memory formation [49–51]. Several plasticity studies in aged animals have reported alterations in activity-dependent forms of synaptic plasticity in the hippocampal CA1 region, which might underlie age-dependent deficits in learning and memory processes [10–12]. Aging causes a deficit in LTP induction, which can be overcome with strong electrical stimulation [46, 52]. In most cases, hippocampal slices from aged animals were used to show abnormalities in the LTP response [10–12, 53]. However, it has become increasingly important to progress electrophysiology research from *in vitro* brain slices to *in vivo* study of the intact brain. As it may be more physiologically representative of the processes occurring in the human brain, accounting for the inputs from different brain regions which may influence the synaptic response. In the present study, we found that aged mice have reduced response, which sustained over the recording period. Decreased LTP with aging was not accompanied by significant alteration in basal synaptic excitability as the I/O curves were not different in young and aged mice.

Ultrastructural and molecular analysis have been used to study synaptic basis of hippocampal memory alterations underlying LTP in young and aged mice during contextual fear memory formation [54]. Unlike young mice, aged mice did not show an increase in mushroom spines and complex PSD, which are characteristics of structural LTP [55, 56]. Although we detected declines in functional LTP in aged mice indicating changes in synapse morphology, there was still a response to HFS suggesting that aged mice could still use NMDA receptor-dependent LTP in hippocampal area CA1. However, NMDA receptor-independent component of LTP has been shown to occur more predominantly in the hippocampus of aged animals and involves high activation of L-type voltage-gated calcium channels (VGCC) [5, 8], likely owing to the increases in density of functional VGCC in CA1 hippocampal neurons during aging [57–59]. In addition, aging is accompanied with Ca^2+^ dysregulation and loss of input specificity in LTP, which may contribute to diminished NMDA-dependent component of LTP [60–62].

### 4.2. Aged Mice Exhibit Deficits in Network Oscillations at the OB Circuit

The olfactory system, which has a high degree of organization and connectivity, is an attractive brain region for neurobiological studies of sensory and cognitive functions in aging [16, 63]. Odor information is encoded by sensory neurons in the olfactory epithelium and then relayed to the olfactory bulb (OB) for processing before distribution to olfactory cortex via the lateral olfactory tract. In the OB region where olfactory information is first processed in the brain, GABAergic inhibitory neurons greatly outnumber principal mitral cells [64], suggesting that odor representations in the olfactory bulb are strongly shaped by local inhibition. The dense connectivity of PV cells with mitral cells [65, 66], and the reciprocal dendro-dendritic signaling between mitral and Fast-spiking cells, indicates that PV+ interneurons play an important role in the processing of sensory information in the olfactory bulb [67].

Rhythmic neural activity has been proposed to play a fundamental role in cognition, and both healthy and pathological aging are characterized by frequency-specific changes in oscillatory activity. Neural activity that oscillates at the gamma rhythm results from the interplay of local inhibitory neurons and excitatory neurons [68, 69], as well as their interactions with local excitatory neurons [70–73].

The firing of fast-spiking PV+ interneurons plays a key role in the generation of gamma oscillations (30–100 Hz), considered as a fundamental mechanism underlying information sensory processing, behavioral control, and memory formation.Olfactory dysfunction has also been reported in aging rodents [17, 18, 69, 74–76]. Age-related degeneration of PV-immunoreactive neurons was observed in the OB of middle-aged and aged dogs [77] and rats [78], likely associated with the rise in the cerebral metabolic rate of oxygen and reduced calcium activity of PV interneurons [79]. Therefore, an alteration of PV interneuron function may be a neurobiological basis underlying impairments of fast network oscillations and thus higher olfactory functions in old adult mice.

### 4.3. Aged Mice Exhibit a Leftward Shift in Network Oscillations at the OB Circuit

Brain aging is markedly characterized by a shift in oscillatory rhythms from higher to lower frequencies, which can be captured by spectral data in higher and slow delta/theta and higher frequencies. The so-called slowing of resting state EEG rhythms observed in humans across physiological and pathological aging has also been observed in aged mice [30]. Some other studies have reported no effect of aging process on the amplitude of hippocampal theta oscillations while decreases in its frequency was observed [80, 81]. Hippocampal theta oscillations play an essential role in learning and memory and much evidence to this theory related theta activity to mnemonic task performance [23, 82], and decline or slowing of the hippocampal theta rhythm in may indicate a decline in learning and memory processes. In the present study, no major alteration was found in theta rhythm in LFP derived from EC and frontal cortical areas; however, a leftward shift in the power towards slow delta oscillations has been found in the OB and hippocampal CA1 area suggesting a decline in odor memory processing in aged animals.

The dynamic interactions between amygdala and hippocampus are critical for emotional memory. Anatomical studies demonstrated large interconnections between the amygdala and the hippocampus as well as with the prefrontal cortex [83–86]. The patterns of oscillatory activity have been studied in relation to fear behavior evoked by conditioned and indifferent sensory stimuli and contexts. Theta synchrony between these structures occurs during fear memory retrieval and may might contribute to improvement in emotional and cognitive processes [87, 88]. Theta synchrony between amygdala and hippocampal CA1 nuclei increases during fear memory retrieval in rodents [86], and the degree of theta synchrony predicts memory performance after fear conditioning [89]. Experience-dependent molecular changes underlying synaptic plasticity during learning and memory can be impaired by aging [90, 91]. In the present work, there was no major changes in the theta or other oscillatory rhythms at the amygdala, and there was no entrainment of the amydala nuclei to theta input as revealed by weak coherence with hippocampus and frontal cortex. This would be compatible with recordings performed under normal conditions where animals were not confronted with conditioned fear stimuli, thus future experiments in aged mice after contextual fear might provide important mechanistic insights regarding oscillatory connectivity the hippocampus and amygdala.

### 4.4. Aged Mice Exhibit Deficits in Network Connectivity

In both humans and rodents, aging is linked to synaptic damage and aberrant functional circuitry leading to impairments in hippocampus dependent learning. Aged rats had lower coherence in theta and gamma frequency oscillations across dorsal CA1 pyramidale and radiatum layers [92]. The reduced coherence associated with pronounced weak strength of the theta-gamma PAC coupling in aged mice animals indicates that gamma is likely to be slower as a result of changes in the response of GABAergic neurons to glutamatergic signals. The neurobiological mechanisms underlying the deficit in high frequency oscillations may be related to age reduction in axon myelination, which may slow the conduction speed observed also in event related potentials [93, 94].

### 4.5. Implication of Current Work for Models of Aging-Related Neurodegeneration

Structural and pathophysiological studies revealed common deficits in the olfactory processing during senescence in humans and rodents, suggesting specific susceptibility of this brain circuit to aging processes [95]. In addition, the OB system appears to be particularly vulnerable to age-related neurodegenerative disease [96–98] and therefore offers a strong predictive utility of olfactory tests for progression from normal aging to MCI and to AD [99]. MCI patients display significant deficits in olfactory identification tests when compared to healthy elderly people [98–100]. At the functional level, late components of the olfactory event-related potential show increased latency with age, suggesting decline in odor processing [101]. Similarly, functional MRI during olfactory identification and discrimination tests demonstrated abnormal blood-oxygen-level-dependent (BOLD) responses in the piriform cortex and frontal and temporal lobes of older subjects [102, 103].

Olfactory dysfunction has also been observed in aging rodents [17, 74–76, 104], likely related to a decline in the density of olfactory receptor neurons [105, 106]. Aged mice showed deficits to learn two-odor discrimination problems for positive reinforcement and failed to show improvement across multiple discrimination problems when compared to young mice [18]. Neuronal recordings from orbitofrontal cortex revealed aberrant responses to odors during reversal tasks in older rats [76], and deficits in olfactory specific learning have been reported in 2-year-old rodents [74, 105], as well as for odor long-term memory, which may be cortical-dependent [105]. Experimental studies have shown a link between gamma oscillations and odor discrimination [107–109]. Accordingly, blockade of gamma oscillations in honeybees resulted in poor performance on discrimination tasks between similar odors [110]. However, increases of gamma oscillations were observed in the OB of rats performing in discrimination tasks [111]. In the present study, aged mice exhibit impairments in high frequency network oscillations, a shift into slow oscillation and deficit in connectivity at the OB circuit, which likely represent neurobiological bases underlying odor deficits olfactory memory deficits. The use of quantitative sleep EEG analysis has been established as a promising biomarker for aging people at risk of cognitive decline [112]. Olfactory decline with normal aging in humans appears to localize to the olfactory epithelium and higher cortical areas and therefore changes in EEG recordings predisposed the early onset of mild cognitive impairment. From the translational perspective, the olfactory bulb and hippocampus were chosen because of their wide connectivity and role in spatial learning, memory, and odor sensory processing, known to be compromised by aging and in age-related neurodegeneration. While basal synaptic activity was not altered, the decline of hippocampal synaptic transmission was only observed in response to HFS. However, the age-dependent alterations in network gamma oscillations and connectivity under spontaneous conditions appeared to be specific to the OB circuit, suggesting the neurophysiological basis of synaptic deficits underlying odor sensory processing. The findings highlight the potential use of LFP quantitative network oscillations and connectivity at the OB neural network as a sensitive electrophysiological marker that will help reveal specific dysfunctional circuits in neurodegeneration studies related to aging.

## Data Availability

All relevant data within the paper are fully available.
